# A look inside Chediak-Higashi syndrome: a microscopic view of the disorder

**DOI:** 10.11604/pamj.2026.53.105.40196

**Published:** 2026-03-02

**Authors:** Suhit Naseri, Samarth Shukla

**Affiliations:** 1 Department of Pathology, Datta Meghe Medical College, Datta Meghe Institute of Higher Education and Research, Wanadongri, Nagpur, India,; 2 Department of Pathology, Jawaharlal Nehru Medical College, Datta Meghe Institute of Higher Education and Research, Sawangi, Wardha, India

**Keywords:** Chediak-Higashi syndrome, genetics, abnormal granules

## Image in medicine

A 3-year-old boy presented with recurrent episodes of lower respiratory tract infections since infancy, along with noticeable hypopigmentation of the skin and silvery hair. He was born to non-consanguineous parents, with no significant perinatal or family history. On examination, the child had pallor and partial oculocutaneous albinism but no organomegaly at presentation. Peripheral blood smear evaluation revealed characteristic giant azurophilic cytoplasmic granules within neutrophils (A), lymphocytes (B, C), and monocytes, suggestive of a lysosomal trafficking defect. These findings, in conjunction with recurrent infections and hypopigmentation, led to a diagnosis of Chediak-Higashi Syndrome. Hematological workup showed mild anemia without evidence of an accelerated phase. Differential diagnoses, including oculocutaneous albinism, Hermansky-Pudlak syndrome, and Griscelli syndrome, were considered; however, the presence of pathognomonic giant granules in leukocytes confirmed the diagnosis. The child was managed with prompt antibiotic therapy for active infection and supportive care. Parents were counselled regarding the genetic basis of the disorder and the risk of progression to the accelerated phase. Hematopoietic stem cell transplantation was discussed as a potential definitive treatment option in severe disease. On short-term follow-up, the child showed improvement in respiratory symptoms and remains under regular monitoring for infectious and hematological complications.

**Figure 1:**
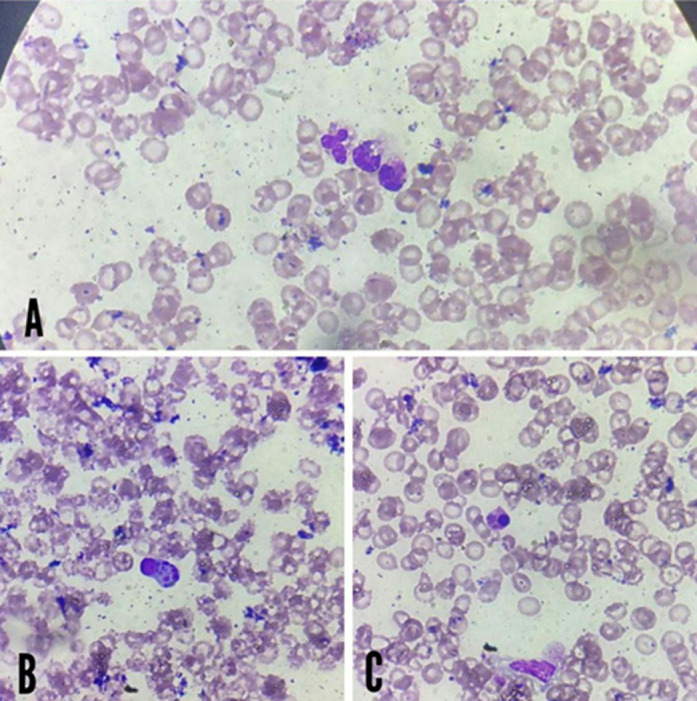
A, B, C) abnormal granules seen in neutrophils and lymphocytes

